# Supplementary material for: A Cost-Effective Nucleic Acid Detection System Using a Portable Microscopic Device
Source: Micromachines (Basel). 2022 May 31;13(6):869. doi: 10.3390/mi13060869 (PMC9227208; doi:10.3390/mi13060869)
Supplement: Supplementary file 1 [file micromachines-13-00869-s001.zip › micromachines-1722174-supplementary figure.pdf]

Supplemental Materials

A Cost-Effective Nucleic Acid Detection System Using a Portable Microscopic Device

Chengzhuang Yu [1], Shanshan Li [1,2,*], Chunyang Wei [1], Shijie Dai [2,*], Xinyi Liang [3] and Junwei Li [3,*]

[1] Hebei Key Laboratory of Smart Sensing and Human–Robot Interactions, School of Mechanical Engineering, Hebei University of Technology, Tianjin 300130, China; 201811201004@stu.hebut.edu.cn (C.Y.); 201811201008@stu.hebut.edu.cn (C.W.)
[2] State Key Laboratory of Reliability and Intelligence of Electrical Equipment, Hebei University of Technology, Tianjin 300130, China
[3] Institute of Biophysics, School of Health Sciences and Biomedical Engineering, Hebei University of Technology, Tianjin 300130, China; 202121103006@stu.hebut.edu.cn
* Correspondence: sli_mems@hebut.edu.cn (S.L.); dsj@hebut.edu.cn (S.D.); junwei_li@hebut.edu.cn (J.L.)

Supplemental Materials List:

Figure S1: The estimation of the field-of-view of our portable microscopic device from a commercial hemocytometer.
Video S1: The operations for the XYZ movement

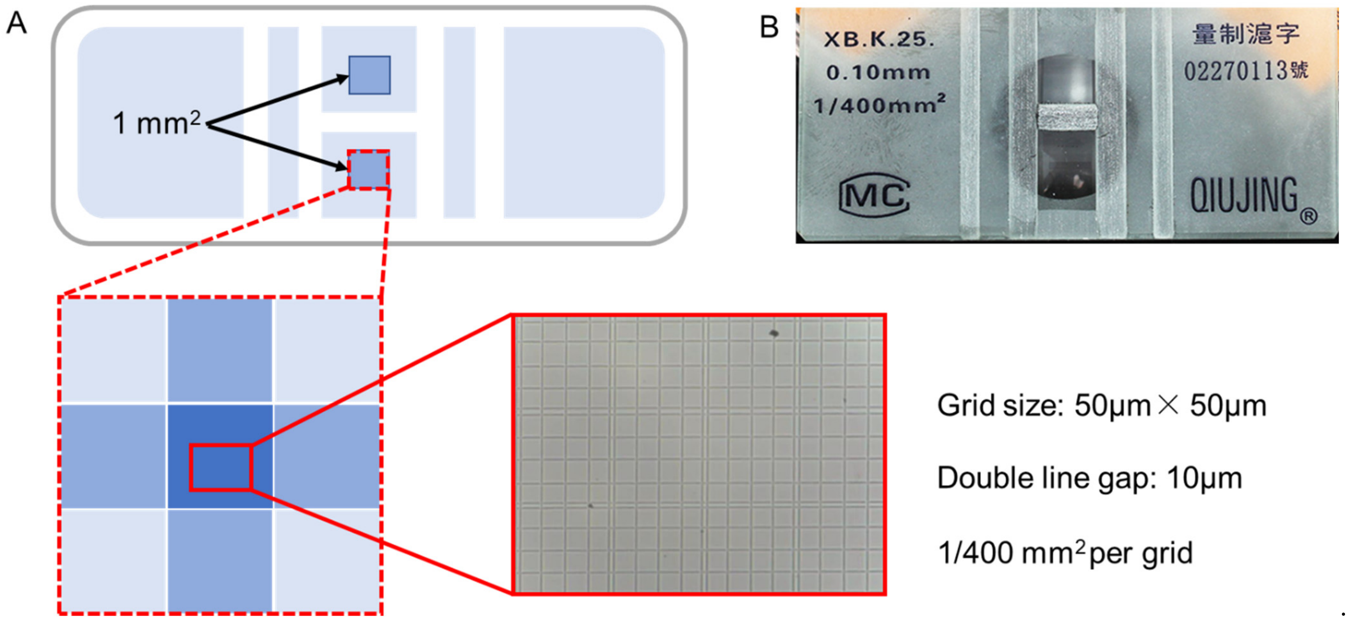

Figure S1. The estimation of the field-of-view of our portable microscopic device from a commercial hemocytometer. (**A**) the meshing grids of the hemocytometer. (**B**) the physical image of the commercial hemocytometer used in this work.
